# Ethical Considerations in Using Brain Stimulation to Treat Eating Disorders

**DOI:** 10.3389/fnbeh.2014.00351

**Published:** 2014-10-09

**Authors:** Kayleigh C. Widdows, Nick J. Davis

**Affiliations:** ^1^Swansea University, Swansea, UK

**Keywords:** TMS, tDCS, neuroethics, safety, anorexia, bulimia

## Introduction

Eating disorders (EDs), such as anorexia nervosa (AN), bulimia nervosa (BN), and binge eating disorder (BED) are characterized by pathological eating behaviors and body image disturbance. These disorders are associated with high levels of mortality and morbidity, as well as significantly impaired quality of life (Arcelus et al., 2011). EDs are often associated with young adulthood, with the disorder typically being first diagnosed when the person is 15–19 years old (Hoek and van Hoeken, 2003; Hudson et al., 2007). Recently, there has been increased interest in the neurobiology of EDs as an insight into the mechanisms of pathological eating behavior, and as a potential avenue for treatment (Kaye et al., 2010).

Brain-based interventions for EDs have in the past involved highly invasive deep-brain stimulation (DBS), in which surgically implanted electrodes deliver electrical pulses to brain areas such as the cingulate cortex (Israel et al., 2010) or the nucleus accumbens (Wu et al., 2013). These surgeries have proved reasonably effective in the small number of reported cases. However DBS has a number of problems that make it less attractive as a treatment option: DBS exposes the person to the risks of surgery; the potential side-effects are more serious; and it is difficult to adjust or to reverse the treatment. For these reasons, there is much interest in a treatment that modulates brain activity but that does not expose the patient to such serious side-effects.

There has recently been an increase in interest in the use of so-called non-invasive brain stimulation to treat EDs. These techniques, principally transcranial magnetic stimulation (TMS) and transcranial direct current stimulation (tDCS), use magnetic or electric fields to transfer energy across the skull, and so to modulate neural activity. Here, we explore the rationale for using TMS/tDCS in EDs, and argue that many ethical and safety issues must be clarified before widespread adoption of these techniques is possible.

## Transcranial Stimulation in EDs

Transcranial magnetic stimulation uses pulsed magnetic fields, which cross the skull and generate electrical effects in nerve cells in the cortex. Two variants of TMS are most likely to show promise in EDs: repetitive TMS (rTMS) uses trains of pulses spaced at 1 Hz, and typically has an inhibitory effect on the brain area being targeted (Rossi et al., 2009); the related technique of theta-burst TMS delivers the pulses more rapidly and in clusters, with the effect on brain tissue being excitatory or inhibitory depending on the parameters used (Huang et al., 2005). tDCS uses small electric currents that cross the skull and induce electric fields on the cortical surface. These fields alter the excitability of the neurons close to the electrodes, with cells close to the positive electrode (anode) becoming slightly more excitable and those near the negative electrode (cathode) reducing in activity. Collectively, these techniques are often referred to as non-invasive brain stimulation, although we have argued that the term “non-invasive” may be inaccurate, and misleading to naïve participants (Davis and van Koningsbruggen, 2013).

Brain imaging has allowed a greater understanding of the neural mechanisms of different EDs to be developed (Kaye et al., 2009; Schäfer et al., 2010). For instance, fMRI studies have found that individuals with AN often have abnormalities in frontal and subcortical areas involved in processing reward learning (Celone et al., 2011). The parietal cortex has also been implicated in maintaining a normal representation of the body (Boghi et al., 2011). The discovery of differences in brain processes between healthy people and those with EDs motivates the use of TMS and tDCS to target cortical regions that may be under- or over-active in EDs. For example, in one study, rTMS over the left dorsolateral prefrontal cortex (DLPFC) in AN patients resulted in reductions in feeling fat and full and in anxiety, but had little impact on mood, tension, or urge to exercise (Van den Eynde et al., 2013). In another study, two people with AN received rTMS to left DLPFC and showed a reduction in symptoms for a short while, although their weight and BMI had worsened on follow-up (McClelland et al., 2013).

However, although brain imaging studies have afforded a better understanding of the neural underpinnings of EDs, our knowledge is still fairly limited and this raises the ethical question about how to justify targeting a specific region for different EDs, when there is little concrete evidence that those regions are definitively involved (Illes et al., 2006).

## Safety and Dosage of Transcranial Stimulation

Transcranial magnetic stimulation and tDCS are relatively safe interventions when used in healthy people and within known safety limits. Known side-effects include headache, mild hearing effects, and skin tenderness. In more serious cases, there may be changes in cortical excitability leading to seizure or to mood changes. Within the established limits of 2 mA for tDCS or 1000 pulses for TMS, these serious side-effects are rare (Bikson et al., 2009; Rossi et al., 2009; Davis et al., 2013). There is however a considerable gap in our knowledge of how TMS and tDCS exert an effect on the brain given a certain level of stimulation, or to put it another way, there is no principled method for setting dosage in TMS/tDCS.

At present, the best method for estimating in advance the appropriate dosage of stimulation to deliver to a person is to create a computational model of the person’s head tissue, and to calculate the transmission of electric or magnetic energy through the different tissue types to a target brain area. These models may also aid in positioning the electrodes or coil for better targeting. For example, modeling of the electric field during tDCS has been used to determine the optimal electrode montage to treat the pain of fibromyalgia (Mendonca et al., 2011), and related approaches have been used to estimate how to set dosage in TMS given variability in scalp-to-cortex distance (Stokes et al., 2005). These approaches are costly and time-consuming since they require access to an MRI facility to scan the person’s head tissue, and finite-element modeling experience to create the head model, and are therefore rarely used in tDCS research as it is often sufficient to use an anatomical landmark for positioning (e.g., Koessler et al., 2009). Recently, some packages have been developed that automate some of these processes (e.g., Truong et al., 2014). Frequently, these packages use a “standard” head model to be representative of a “normal” adult head. We would urge caution in generalizing these models to people with EDs.

Eating disorders are typically a disorder of young adulthood (Hoek and van Hoeken, 2003; Hudson et al., 2007). We have previously noted that stimulation protocols that are effective and tolerable in adults may not necessarily be safe or tolerable in younger people (Davis, 2014). There are qualitative differences in the head tissue of younger people that make the standard models inapplicable. Specifically, the smaller size of the head and the more efficient flow of current across the skull mean that a specific dose of stimulation will have a larger effect on the brain of a child than it will on the brain of an adult (Kessler et al., 2013).

As well as the problems of stimulating dosage in younger people, people with EDs present additional challenges. Fat deposits in the head add to the insulating qualities of the various tissue types, meaning that lower levels of fat will lead to a higher transfer of energy to the brain surface. A recent modeling study suggested that excess fat due to obesity made the tDCS-induced electric field less easy to predict (Truong et al., 2013); however, the equivalent study for lower-than-normal cutaneous fat has not been performed. It is also worth noting that demineralized bone has greater electrical permittivity than normal bone, suggesting that stimulation transfer is more efficient in people with a less mineral-rich diet (Ivancich et al., 1992). A further problem that complicates the use of standardized head models is the altered cortical folding in people with EDs. People with BN show characteristic patterns of enlargements and deformations of cortical areas, particularly around the prefrontal cortex and the occipital pole (Marsh et al., in press). These morphological differences mean that TMS is more or less effective in these areas (Stokes et al., 2005), or that the tDCS-induced field is distributed differently (Miranda et al., 2006). Taken together, these differences between the head tissue of healthy people and people with EDs imply that clinicians and researchers should exercise caution in setting the dose of any TMS/tDCS-based intervention.

In addition to the above factors, it should be noted that the efficacy of brain stimulation is frequently state-dependent (e.g., Silvanto et al., 2008). The brain responds rapidly to the person’s nutritional state (Streitburger et al., 2012). Studies analyzing brain morphology in EDs require careful control of the person’s nutritional state in order to make group comparisons (e.g., Frank et al., 2013a,b). We therefore recommend that future studies involving people with EDs should control and report the nutritional state of the participants. Future clinical applications of TMS/tDCS should also be mindful of the state-dependence of brain stimulation, which may include medicative state as well as nutritative (Davis et al., 2013).

## Ethical Issues in Treating EDs

A recent article explored the ethical issues involved in brain stimulation treatments for AN (Coman et al., 2014). They focused on the principles of beneficence (equivalent to efficacy of treatment), non-maleficence (avoidance of side-effects), respect for autonomy (informed consent and capacity for consent), and justice (fair access to treatment). Of these principles, beneficence is the least easy to assess due to the small number of studies that have been reported in this area; however, there appear to be grounds for optimism. Importantly, researchers and clinicians should adhere to good research practices in developing well-controlled experiments and in reporting all experimental results (Davis et al., 2013). A complicating factor in assessing efficacy is the ethical problem of including a no-treatment group in any trial, where doing nothing may seriously imperil the health of the patient. Access to treatment is not currently a pressing issue, as the necessary equipment is confined to specialist labs and clinics; however, if the techniques become more widespread there may arise the need for clear guidelines to prioritize treatment for those with most to gain. However, a related issue is the balance between protecting individual rights, and developing and testing treatments for the good of a wider population; although there is no clear answer to this dilemma, we can at least ensure that individuals understand the different levels of risks and benefits before enrolling in a trial. As a guiding principle, we argue that the safety of vulnerable participants should be paramount, and that trials and treatments should minimize both the number of participants (while still retaining statistical power) and the dose delivered to each participant (Davis et al., 2013).

Non-maleficence should be the overriding concern of any clinician when prescribing a treatment. This article is an attempt to enumerate a number of factors that make it difficult to assess safety, and therefore, maleficence, in relation to TMS/tDCS in people with EDs. We therefore suggest that it is a clinician’s and an experimenter’s duty to critically evaluate the published reports of TMS/tDCS in EDs to judge the safety of a technique in relation to a particular patient. The gaps in our knowledge about the effects and side-effects of stimulation mean that a person’s ability to give fully informed consent to treatment is limited by the clinician’s own knowledge. This and other factors related to assumptions about the ability of a person with ED to hold the capacity for informed consent (Blinder et al., 2006; Abbate-Dago et al., 2013; Coman et al., 2014), mean that particular care must be taken to respect the autonomy of a person considered for treatment with TMS/tDCS.

## Conclusion

In this paper, we have argued that treatment of EDs with transcranial stimulation introduces a number of ethical and safety issues that require some examination. These issues are all the more pressing given the rise in awareness and availability of brain stimulation to lay people. For example, it is possible to build an electronic circuit to deliver tDCS for very little cost, which has given rise to a movement in so-called “DIY-tDCS” (Fitz and Reiner, 2013). We as researchers and clinicians therefore have a duty to deal honestly with the technical and ethical problems that arise in a rapidly developing field.

We have identified a number of key questions about TMS/tDCS that should be addressed. Primarily, it will be crucial to understand how to set the appropriate dose of stimulation for a given patient, brain target, and desired effect. This is true for all brain stimulation, but particularly in the case of people with EDs who may have qualitative differences in anatomy compared to healthy people. It is likely that individual MRI-derived head models will prove to be the most effective means of dose-setting. We also suggest that clinicians and researchers are encouraged to report fully the results of every trial. This is an element of good scientific practice that is difficult to police; however, the benefits are clear in terms of reducing wasted time and resources in repeating trials, and in minimizing exposure of patients to potentially harmful treatments (Davis et al., 2013). We recommend that scientists and clinicians proceed cautiously when designing protocols, and do not translate protocols designed for use in healthy adults directly to use in people who are younger or who may have altered cranial anatomy. If MRI-based modeling is not available, we recommend reducing stimulation intensity for use in people with EDs.

The relative cheapness and the safety of brain stimulation techniques such as TMS and tDCS make it likely that they will continue to develop as standalone or adjunctive treatments for people with EDs. We argue that wider use of these treatments must be combined with a deeper understanding both of the neurophysiological mechanisms of the stimulation, and of the neural bases of the disorders themselves. We are optimistic that brain stimulation will be of great benefit to people who suffer from these elusive and devastating disorders.

## Conflict of Interest Statement

The authors declare that the research was conducted in the absence of any commercial or financial relationships that could be construed as a potential conflict of interest.
